# The effect of the histone deacetylase inhibitor M344 on BRCA1 expression in breast and ovarian cancer cells

**DOI:** 10.1186/1475-2867-11-29

**Published:** 2011-08-19

**Authors:** Johanne I Weberpals, Anna M O'Brien, Nima Niknejad, Kyla D Garbuio, Katherine V Clark-Knowles, Jim Dimitroulakos

**Affiliations:** 1 Division of Gynaecologic Oncology, The Ottawa Hospital, 501 Smyth Road, Ottawa, K1H 8L6, Canada; 2 Centre for Cancer Therapeutics, Ottawa Hospital Research Institute, 501 Smyth Road, Ottawa, K1H 8L6, Canada; 3 Department of Medicine, the University of Ottawa, 451 Smyth Road, Ottawa, K1H 8M5, Canada; 4 Department of Biochemistry, the University of Ottawa, 451 Smyth Road, Ottawa, K1H 8M5, Canada

## Abstract

**Background:**

The inhibition of Breast Cancer 1 (BRCA1) expression sensitizes breast and ovarian cancer cells to platinum chemotherapy. However, therapeutically relevant agents that target BRCA1 expression have not been identified. Our recent report suggested the potential of the histone deacetylase (HDAC) inhibitor, M344, to inhibit BRCA1 expression. In this study, we further evaluated the effect of M344 on BRCA1 mRNA and protein expression, as well as its effect on cisplatin-induced cytotoxicity in various breast (MCF7, T-47D and HCC1937) and ovarian (A2780s, A2780cp and OVCAR-4) cancer cell lines.

**Results:**

With the addition of M344, the platinum-sensitive breast and ovarian cancer cell lines that displayed relatively high BRCA1 protein levels demonstrated significant potentiation of cisplatin cytotoxicity in association with a reduction of BRCA1 protein. The cisplatin-resistant cell lines, T-47D and A2780s, elicited increased cytotoxicity of cisplatin with M344 and down regulation of BRCA1 protein levels. A2780s cells subjected to combination platinum and M344 treatment, demonstrated increased DNA damage as assessed by the presence of phosphorylated H2A.X foci in comparison to either treatment alone. Using Chromatin Immunoprecipitation, A2780s and MCF7 cells exposed to M344 alone and in combination with cisplatin, did not demonstrate enhanced acetylated Histone 4 at the *BRCA1 *promoter, suggesting an indirect effect on this promoter.

**Conclusions:**

The enhanced sensitivity of HDAC inhibition to platinum may be mediated through a BRCA1-dependent mechanism in breast and ovarian cancer cells. The findings of this study may be important in the future design of clinical trials involving HDAC inhibitors using BRCA1 as a tumour biomarker.

## Background

Epithelial ovarian cancer (OC) is the fifth leading cause of cancer death in women and the most lethal gynecologic malignancy [1]. In spite of aggressive surgical cytoreduction and combination platinum/paclitaxel chemotherapy, over 75% of women with stage III/IV disease will relapse and succumb to their disease. Resistance to platinum-based therapy is a primary obstacle in the management of advanced OC and novel therapies are required to enhance platinum chemotherapy and to improve prognosis. Hereditary mutations in the Breast Cancer 1 (*BRCA1) *tumor suppressor gene are associated with a significant risk of developing breast and OC [2,3]. Although somatic mutations in *BRCA1 *are uncommon in sporadic OC, BRCA1 dysfunction is frequently observed [4]. Silencing of *BRCA1*, through promoter methylation, decreased expression through gene deletion (loss of heterozygosity), or dysregulation of related genes in the Fanconi anemia/BRCA1 pathway, is believed to be important in the pathogenesis of a significant proportion of sporadic tumors [5].

Preclinical work has shown that the level of BRCA1 protein expression correlates with chemosensitivity [6], and recent clinical data supports that BRCA1-deficient OC patients have a better prognosis [4,7]. Low BRCA1 protein and mRNA expression has also been associated with improved survival in breast cancer [8] and non-small cell lung cancer [9]. The improved outcome in BRCA1-deficient tumors is believed to be due, in part, to an increased sensitivity to DNA damaging chemotherapeutics, such as cisplatin [5]. Cells that lack BRCA1 have a deficiency in the repair of double strand breaks by the conservative mechanism of homologous recombination (HR) [10]. As a result, these cancer cells are reduced to using error-prone pathways thereby leading to genomic instability and enhanced cisplatin cytotoxicity. Thus, BRCA1 has been regarded as a rational therapeutic target to help overcome platinum resistance in advanced and recurrent OC. However, in an era of evolving molecular inhibitors, new therapeutic strategies merit consideration.

The interaction between histone acetyl transferases and histone deacetylase (HDAC) enzymes modulates chromatin structure and transcription factor accessibility, resulting in changes in gene expression [11]. Inhibitors of HDAC have pleiotropic effects on cell cycle arrest, apoptosis, differentiation and inhibition of growth and angiogenesis [12,13], and have emerged as promising new therapeutic agents in multiple cancers, including those resistant to standard chemotherapy. Class I HDAC isoforms are expressed at significantly higher levels in OC compared to normal ovarian tissue [14], and various HDAC inhibitors can prevent the growth of OC cancer cells both *in vitro *and *in vivo *[15,16]. Furthermore, HDAC inhibitors promote the accumulation of acetylated histones, resulting in a more relaxed chromatin structure, with areas of loosely compacted, and hence, more transcriptionally active chromatin that is more prone to DNA double strand breaks [17]. In this regard, HDAC inhibitors have also demonstrated in the preclinical setting the ability to potentiate the effects of DNA-damaging agents, such as ionizing radiation and several chemotherapeutic agents such as topoisomerase inhibitors, and platinum compounds [18]. This suggests that HDAC inhibitors have synergistic potential to enhance the treatment of recurrent OC. The evaluation of HDAC inhibitors in phase I/II clinical trials, either as a single agent or in combination with standard cytotoxic chemotherapy, is ongoing in a wide range of malignancies including OC [16,19].

Targeting BRCA1 as a therapeutic strategy merits further study in the management of BRCA1-associated malignancies such as breast and OC. The potent HDAC inhibitor, M344, a synthetic amide analog of trichostatin A, has demonstrated growth inhibition, cell cycle arrest and apoptosis in human endometrial and OC cells [20]. M344 is structurally similar to SAHA (vorinostat), which was approved for the treatment of cutaneous T-cell lymphoma [21]. Our group has recently shown that M344 sensitizes A2780 OC cells to platinum by decreasing the mRNA and protein expression of BRCA1 [7]. Further validation is required to confirm HDAC inhibition on BRCA1 and to explore potential mechanisms of M344 as a targeted agent of BRCA1. In this study, we further evaluate the effect of the combination of M344 and cisplatin on BRCA1 mRNA and protein expression and on cisplatin sensitivity in various breast and OC cell lines.

## Material and methods

### Cell Culture

The A2780s and A2780cp cell lines were kindly provided by Dr. B. Vanderhyden (Ottawa Hospital Research Institute, Ottawa, ON, CAN), and the T-47D and OVCAR-4 cell lines were donated by Dr. J. Bell (Ottawa Hospital Research Institute). MCF7 and HCC1937 were purchased from the American Type Culture Collection (Rockville, MD). All cell lines were maintained in Dulbecco's-MEM (Media Services, Ottawa Hospital Regional Cancer Centre, ON, CAN) supplemented with 10% fetal bovine serum (Wisent Inc., St-Bruno, QC, CAN) and 100 μg/ml penicillin-streptomycin (Invitrogen, Carlsbad, CA). Unless otherwise described, cells were treated for 24 hrs with 2 μg/ml cisplatin (provided by the pharmacy at the Ottawa Hospital Regional Cancer Centre) alone, and in combination with the HDAC inhibitor M344 (Biovision, Mountain View, CA) at concentrations of 0.5, 1.0, or 5.0 μM. Phase contrast images were collected using the 10 × objective of an Eclipse TE2000-U (Nikon Canada Inc., Mississauga, ON, CAN).

### Western Blotting

Protein samples were collected in RIPA buffer (50 mM Tris-Cl pH 7.5, 150 mM NaCl, 1 mM EDTA, 1% Triton X-100, 0.25% sodium deoxycholate, 0.1% SDS) containing 1X Protease Inhibitor Cocktail (Sigma-Aldrich, St-Louis, MO) and protein content was quantified using a commercially available protein assay (BCA Protein Assay Kit, Pierce, Rockford, IL) and a Biomate3 Spectrophotometer (Thermo Fisher Scientific, Waltham, MA). Samples were separated on 8-12% SDS polyacrylamide gel and transferred to a PVDF membrane (Immobilon-P, Millipore, Billerica, MA). Blocking was carried out with 5% milk in Tris-buffered saline with Tween-20 (TBS-T). For all subsequent immunoblotting, antibodies were diluted to the appropriate concentration in 5% milk in TBS-T. Blots were incubated with the following primary antibodies for 1 hr at room temperature or overnight at 4°C: mouse-anti BRCA1 (1:200, D-9, Santa Cruz, Santa Cruz, CA), rabbit-anti acetylated Histone 4 (acetyl H4) (1:1000, Upstate Cell Signaling, Lake Placid, NY), and mouse-anti actin (1:5000, Sigma-Aldrich). Following 3 washes in TBS-T, blots were incubated with the appropriate horseradish peroxidase (HRP)-labeled secondary antibody (goat-anti-rabbit-HRP, goat-anti-mouse-HRP, 1:5000, Jackson ImmunoResearch, West Grove, PA) for 1 hr at room temperature. The chemiluminescent substrate used was Supersignal West Pico (Pierce) and the visualization of the protein bands was performed using the GeneSnap image acquisition system followed by densitometry analysis with the GeneTools software (Syngene, Frederick, MD).

### RNA isolation and reverse transcriptase polymerase chain reaction (RT-PCR)

Total RNA was extracted from cell lines in sub-confluent 10 cm dishes using the RNeasy^® ^kit (Qiagen, Germantown, MD). RNA concentration was quantified using a NanoDrop ND-1000 spectrophotometer (Thermo Scientific Inc, Wilmington, DE). Total RNA (1 μg) was reverse-transcribed. The Applied Biosystems AB 7500 Real-Time PCR system (Applied Biosystems, Foster City, CA) was used to detect amplification. A real-time PCR reaction was carried out in a total volume of 25 μl that contained 2.5 μl of synthesized cDNA, 1.25 μl of TaqMan Gene Expression Assay Primer/Probe (20X) (Applied Biosystems, BRCA1, HS00173233), 12.5 μl of TaqMan Universal PCR Master Mix (2X) (Applied Biosystems) and 8.75 μl of RNase-free water for BRCA1 expression. GAPDH (Applied Biosystems, HS4333764-F) was used as an endogenous control. Amplification conditions were 95°C for 5 min, 40 PCR cycles at 95°C for 15 sec, and 60°C for 1 min. Three independent reactions from separate RNA extractions were used to determine the average RNA expression and a standard error for each treatment condition.

### Cell Viability Assay

Cell viability was measured by the methylthiazolyldiphenyl-tetrazolium bromide (MTT) rapid colorimetric assay. Approximately 4,500 cells were seeded into each well of a 96-well flat bottom plate. The cells were incubated overnight to allow for cell attachment. Cells were then treated with cisplatin in concentrations of 0-8 μg/ml alone or in combination with 1 μM of the HDAC inhibitor, M344. Forty eight hours following treatment, 42 μl of a 5 mg/ml MTT substrate (Sigma-Aldrich) solution in phosphate buffered saline (PBS) was added and incubated for up to 4 hrs at 37°C. The resulting violet formazan precipitate was solubilized by the addition of 82 μl of a 0.01 M HCl/10% SDS (Sigma-Aldrich) solution and plates were incubated overnight at 37°C. The plates were then analyzed on an MRX Microplate Reader (Dynex Technologies, Chantilly, VA) at 570 nm to determine the optical density of the samples.

### Flow Cytometric Analysis of Apoptosis

Cells treated for 24 hrs in 10 cm dishes were fixed in 80% ethanol for 1 hr. Cells were then washed with PBS and resuspended in staining buffer (0.2% Triton X-100, 1 mM EDTA in PBS, pH7.4), containing 25 μg/ml propidium iodide (Sigma-Aldrich) and 100 μg/ml RNaseA (Sigma-Aldrich). Cells were incubated with staining buffer in the dark for 1 hr prior to DNA quantification by the Coulter Epics XL flow cytometer (Beckman Coulter, Fullerton, CA). Data analysis was performed using ModFit *LT *(Verity Software House Inc., Topsham, ME).

### Immunofluorescence

Cells were fixed on gelatin-coated coverslips in cold methanol at -20°C for 1 hr, followed by 3 washes in 1 × PBS. The cells were then permeabilized via incubation with 0.2% Triton-X-100 in PBS for 10 min, followed by 3 washes in PBS. Blocking was carried out for 30 min at room temperature with 5% normal goat serum in PBS. Cells were incubated with mouse anti-H2A.X (ser139) (1:100 in PBS, Millipore) for 1 hr, followed by 3 PBS washes. Secondary antibody, anti-mouse Alexa Fluor 488, (1:400 in PBS, Invitrogen) was applied for 1 hr, followed by 3 washes in PBS. Following a rinse with ddH_2_O, coverslips were mounted on glass slides using Vectashield mounting medium with DAPI (Vector Laboratories, Burlington, ON, Canada). Fluorescence was assessed using the Axioskop 2 MOT microscope (Carl Zeiss MicroImaging, Thornwood, NY).

### Flow Cytometric Analysis of γ-H2A.X Expression

Following treatment, cells were trypsinized, washed in PBS and fixed on ice with 1% paraformaldehyde for 15 min. After centrifugation, the cell pellet was resuspended in 500 μl of PBS and transferred to a tube containing 4.5 ml of cold 70% ethanol and kept at -20°C for a minimum of 2 hrs. Cells were centrifuged and then washed twice in BSA-T-PBS (1% bovine serum albumin and 0.2% Triton-X-100 in 1 × PBS). Following the second wash, the cell pellet was resuspended in BSA-T-PBS containing mouse anti-gamma H2A.X (ser139) (Millipore) primary antibody at 1:100 and incubated overnight at 4°C. Cells were then washed once in BSA-T-PBS and resuspended in BSA-T-PBS containing anti-mouse Alexa Fluor 488 (Invitrogen) secondary antibody at 1:400 and incubated at room temperature in the dark for 1 hr. Cells were washed once in BSA-T-PBS and resuspended in PBS containing 50 μg/ml propidium iodide (Sigma-Aldrich) and 5 μg/ml RNAse A (Sigma-Aldrich). Cells were analyzed on a Coulter Epics XL flow cytometer (Beckman Coulter) and the resulting data was assessed using ModFit software (Verity Software House).

### Chromatin Immunoprecipitation (ChIP) Assay

Cells were fixed in 1% formaldehyde (BDH, VWR International, Mississauga, ON, CAN) for 20 min at room temperature. Fixation was stopped by quenching with 2.5 mM glycine solution to a final concentration of 200 mM for 5 min. Cells were then washed twice with ice-cold PBS and harvested in 1 ml cold PBS by centrifugation for 5 min at 5,000 rpm. The pellet was resuspended in 90 μl lysis buffer (50 mM Tris-HCl pH 8.0, 10 mM EDTA pH 8.0, 1% SDS) supplemented with 1X Protease Inhibitor Cocktail (Sigma-Aldrich), 1 mM 1,4-dithio-DL-threitol (DTT) (Sigma-Aldrich), and 1 mM phenylmethylsulfonyl fluoride (PMSF) (Sigma-Aldrich). The lysates were sonicated using a Sonicator 3000 (Misonix, Farmingdale, NY) to shear DNA to an average size of 300 to 1000 base pairs and then cleared of debris by centrifugation at 14,000 rpm for 15 min. Input controls were removed from each sample and stored at -20°C.

The sonicated lysates were diluted 10-fold with dilution buffer (20 mM Tris-HCl pH 8.0, 150 mM NaCl, 2 mM EDTA pH 8.0, 1% Triton X-100), supplemented with 1X Protease Inhibitor Cocktail, 1 mM DTT and 1 mM PMSF, and immunoprecipitated by overnight rotation at 4°C with rabbit-anti acetyl H4 (1:200, Millipore) primary antibody. Negative controls were incubated in the absence of primary antibody. Immune complexes were collected by 2 hr rotation at 4°C with the addition of 40 μl of protein A agarose/salmon sperm DNA 50% slurry (Millipore) to both positive samples and negative controls. The beads were pelleted gently by centrifugation for 1 min at 3,000 rpm at 4°C and washed with 1 ml of the following buffers by rotation for 10 min at 4°C: Buffer A (low salt; 0.1% SDS, 1% Triton X-100, 20 mM Tris-HCl pH 8.0, 2 mM EDTA pH 8.0, 150 mM NaCl) once, Buffer B (high salt; 0.1% SDS, 1% Triton X-100, 20 mM Tris-HCl pH 8.0, 2 mM EDTA pH 8.0, 500 mM NaCl) once, Buffer C (1% NP-40, 1% sodium deoxycholate, 20 mM Tris-HCl pH 8.0, 1 mM EDTA pH 8.0, 0.25 M LiCl) once and TE washing buffer (10 mM Tris-HCl pH 8.0, 1 mM EDTA pH 8.0) twice. All antibody complexes were eluted with 400 μl freshly prepared elution buffer (1% SDS, 100 mM NaCHO_3_) by rotating at room temperature for 30 min. Cross-links were reversed by overnight incubation with 100 μg proteinase K (Roche Diagnostics, Laval, QC, CAN) at 65°C.

DNA was purified using a QiaQuick PCR Purification Kit (Qiagen) according to the manufacturer's instructions. Quantitative PCR was performed using a Roche LightCycler Version 3 (Roche Diagnostics) for 40 cycles of amplification. The binding of acetyl H4 to the BRCA1 proximal promoter region was determined using the following primer pair: forward TTTCCTTTTACGTCATCCGGG and reverse GCTAAGCAGCAGCCTCTCAGA [22]. PCR products were resolved on 1.6% agarose gels.

## Results

### Expression of BRCA1 in a panel of breast and ovarian cancer cell lines

**Figure 1:**
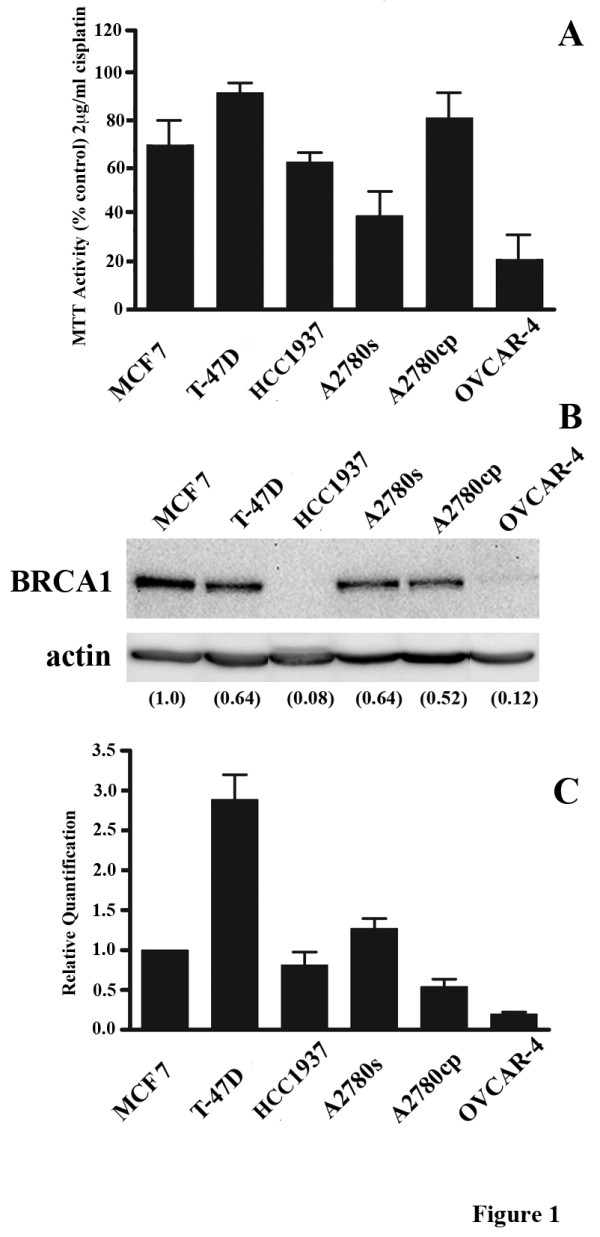
**Cisplatin sensitivity and BRCA1 expression in the breast and ovarian cancer derived cell lines evaluated in this study**. **A) **MTT cell viability assays comparing the responses of a panel of cell lines to 2 μg/ml cisplatin. Cell viability was assayed with the activity of untreated cells taken to be 100%. Values represent the mean +/- SEM of three separate experiments. **B) **Western blot analysis of basal expression levels of BRCA1 protein in a panel of cell lines. Actin was employed as a loading control. Numbers indicate protein densitometry readings with MCF7 used as the calibrator and set to 1.0. The experiment was repeated with similar results. **C) **Basal levels of BRCA1 mRNA analyzed by RT-PCR. Relative expression for each cell line was calculated following normalization to GAPDH levels and then further normalized to MCF-7 for ease of comparison and expressed as the mean +/- SEM of three separate experiments.

Three breast cancer cell lines (MCF7, T-47D, and HCC1937) and three OC cell lines (A2780s, A2780cp, and OVCAR-4) were chosen for analysis due to their varying degree of sensitivity to cisplatin treatment (Figure 1A). Consistent with other reports, T-47D and A2780cp demonstrated cisplatin resistance, whereas MCF7, HCC1937, A2780s, and OVCAR-4 displayed a range of sensitivity to cisplatin treatment [23,24]. The basal level of BRCA1 protein expression was analyzed by Western blot (Figure 1B). MCF7 displayed the most significant level of BRCA1 protein expression of the breast cancer cell lines and was assigned a value of 1.0. As expected, HCC1937 cells, which harbor the germ line BRCA1 frame shift mutation 5382insC, leading to a premature stop codon and a truncated non-functional protein [25], did not display detectable BRCA1 protein. A2780s cells expressed the highest level of BRCA1 protein of the OC cell lines, but only slightly more than their cisplatin-resistant counterpart, A2780cp. All cell lines were evaluated by RT-PCR for BRCA1 mRNA expression with varying levels shown (Figure 1C). HCC1937 cells demonstrated detectable levels of BRCA1 mRNA, albeit lower than the other breast cancer cell lines examined, which is in keeping with the previous observation that tumors from germ line mutation carriers express mRNA levels lower than in sporadic tumors [26]. Overall, variable levels of BRCA1 mRNA and protein were detected in the ovarian and breast cancer cell lines analyzed which is consistent with the range of expression levels previously observed in ovarian and breast tumor specimens [4,7,27].

### M344 reduces BRCA1 mRNA and protein expression in breast and OC cell lines

**Figure 2:**
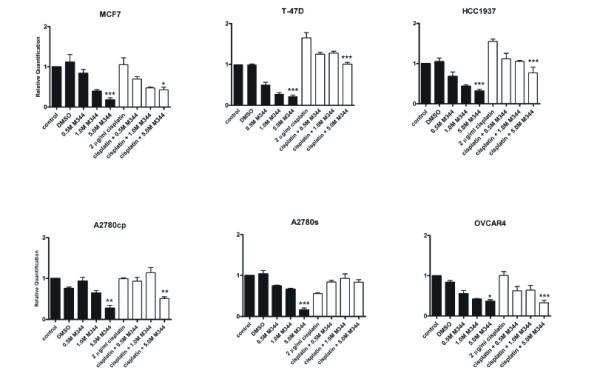
**Effect of M344 and cisplatin treatments on the levels of BRCA1 mRNA in our panel of cell lines**. BRCA1 mRNA levels were analyzed by RT-PCR following 24 hrs of treatment with 0.5, 1.0, or 5.0 μM M344 either alone or in combination with 2 μg/ml cisplatin. DMSO is the solvent control for the M344 treatment and 2 μg/ml cisplatin is the control for the combination. Values represent the mean +/- SEM of three separate experiments. Statistically significant differences, when present, between 5 μM M344 and DMSO control or 2 μg/ml cisplatin + 5 μM M344 and 2 μg/ml cisplatin alone are indicated by * where P < 0.05, ** where P < 0.01, and *** where P < 0.001.

BRCA1 mRNA levels were determined by RT-PCR following exposure to increasing concentrations of the HDAC inhibitor M344 alone and in combination with cisplatin in all 6 cell lines evaluated in this study (Figure 2). With increasing concentrations of M344, there was a dose dependant decrease in BRCA1 mRNA and treatment with both 1 and 5 μM concentrations of M344 resulting in a significant decrease in BRCA1 expression in all cell lines examined. M344 in combination with cisplatin led to a decrease in BRCA1 mRNA expression as compared to cisplatin treatment alone in all cell lines with the exception of A2780s, which is recognized as having potent cytotoxicity to cisplatin.

**Figure 3:**
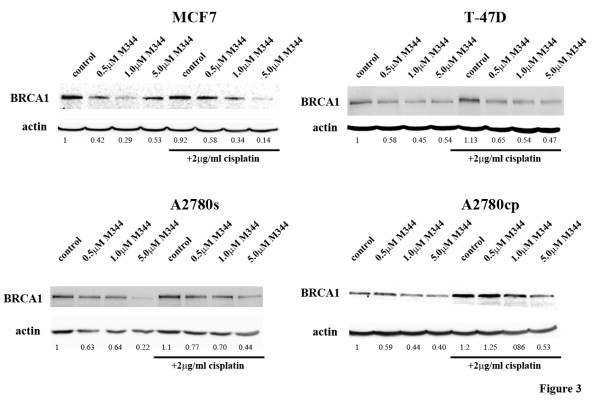
**Effect of M344 and cisplatin treatments on the levels of BRCA1 protein expression in our panel of cell lines**. Western blot analysis of BRCA1 following 24 hrs of treatment with 0.5, 1.0, or 5.0 μM M344 either alone or in combination with 2 μg/ml cisplatin. DMSO is the solvent control for the M344 treatment and 2 μg/ml cisplatin is the control for the combination treatment. The experiment was repeated with similar results. Densitometry readings are normalized to the DMSO solvent control.

The effect on BRCA1 protein expression of M344 alone, and in combination with cisplatin, was assessed by Western blot analysis (Figure 3). Since OVCAR-4 has no measurable BRCA1 protein and HCC1937 has a truncated labile protein, these two cell lines were excluded from this analysis. Of the four remaining cell lines, BRCA1 protein levels decreased with increasing dose of M344. In the MCF7 cell line, BRCA1 was down regulated at physiological doses of M344 (0.5 μM and 1.0 μM) but M344 does not have the same inhibitory effect on BRCA1 at the 5.0 μM dose. Co-treatment with cisplatin and increasing concentrations of M344 reduced BRCA1 protein levels in all breast and ovarian cell lines examined.

### M344 enhances cisplatin sensitivity and increases apoptosis in breast and OC cells

**Figure 4:**
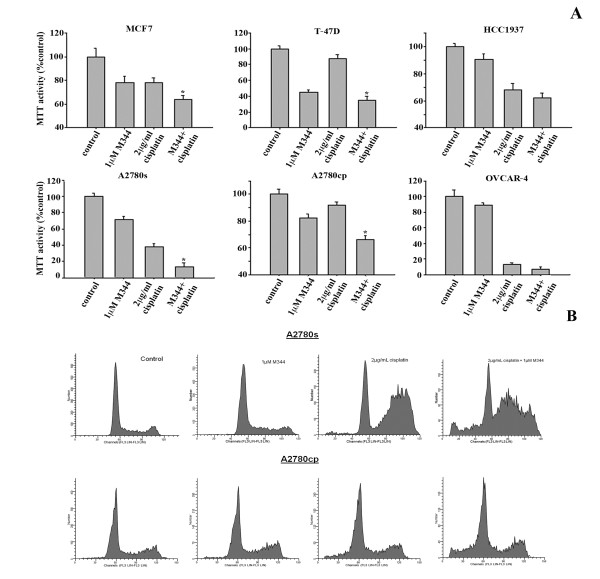
**Analysis of the cytotoxic and apoptotic effects of the combination of M344 and cisplatin inn this cell line panel**. **A) **MTT cell viability assays comparing the responses of a panel of cell lines to 1 μM M344 alone, 2 μg/ml cisplatin alone or both treatments in combination for 48 hrs. Cell viability was assayed with the activity of untreated cells taken to be 100%. Values represent the mean +/- SEM of three separate experiments. Differences between treatment with cisplatin alone versus treatment with the cisplatin and M344 combination were analyzed using paired T-test analyses. * indicates a significant difference where P < 0.001. **B) **Percentage of apoptotic cells in the A2780s cell line following 24 hrs of treatment and A2780cp cell line following 48 hrs treatment with 1.0 μM M344 alone or in combination with 2 μg/ml cisplatin as assessed by flow cytometry. The experiment was done in three replicates with similar results.

The MTT assay was employed to determine the effects on cell viability following treatments with M344 (1 μM, 24 hrs) alone and in combination with cisplatin (2 μg/ml, 24 hrs) (Figure 4A). Of interest, the BRCA1 expressing cell lines (MCF7, T47D, A2780s, A2780cp) demonstrated co-operative cytotoxicity with M344 and cisplatin combination treatments (P < 0.001, cisplatin treatment compared to combination). However, discernable effects on cytotoxicity with this combination treatment were observed in the BRCA1-deficient cells, HCC1937 and OVCAR4. Among the cisplatin-resistant cell lines (T-47D and A2780cp), as expected, there was little effect on cell death with the addition of 2 μg/ml cisplatin. The addition of the HDAC inhibitor resulted in greater overall cytotoxicity and proved to be more effective than cisplatin treatment alone (P < 0.001). Thus, co-treatment with M344 was able to potentiate the effects of cisplatin in breast and OC cells coincident with the ability of M344 to target BRCA1 expression.

To assess the therapeutic effect on apoptosis, two OC cell lines (A2780s and A2780cp) were treated with M344 and cisplatin, alone or in combination, and subjected to flow cytometric analysis (Figure 4B). Treatment with HDAC inhibitor did not cause a marked increase in apoptosis versus control cells, while cisplatin treatment displayed evidence of S/G2 phase arrest in the cisplatin sensitive A2780s cell line. The combination of M344 and cisplatin displayed an apoptotic response as demonstrated by the emergence of a sub-G1 peak characteristic of the nuclear and cellular fragmentation associated with this mode of cell death.

### Co-treatment with the HDAC inhibitor M344 enhanced cisplatin-induced γH2A.X foci formation

**Figure 5:**
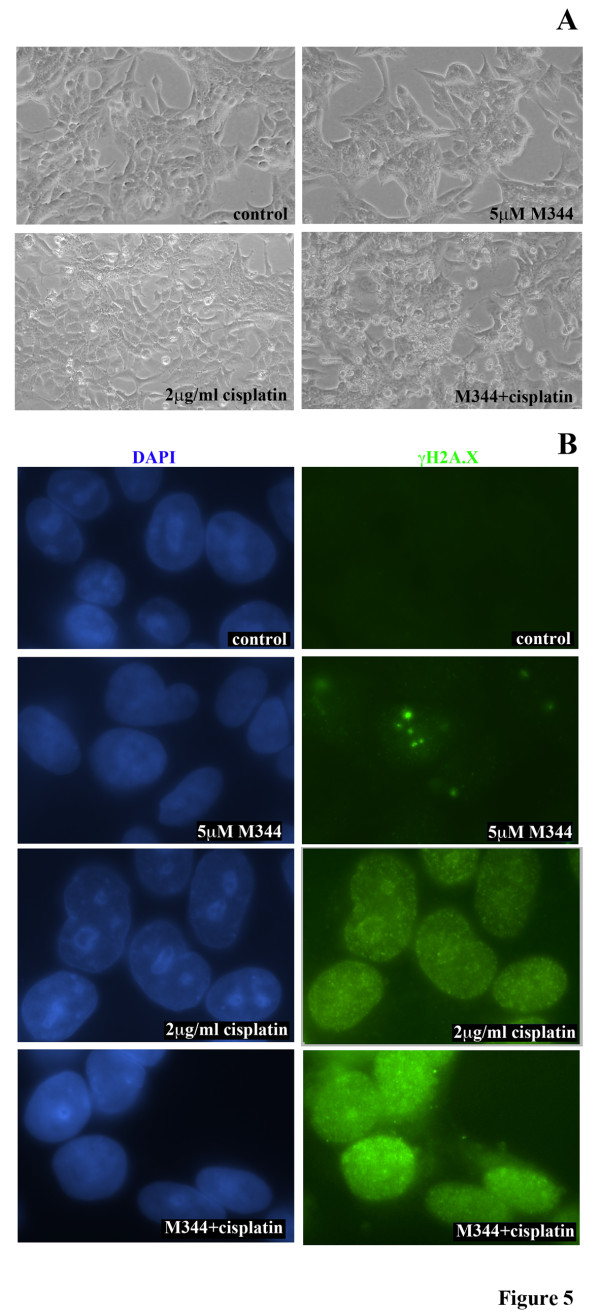
**Evaluating the effect of M344, cisplatin and their combination on the formation of γH2A**.X foci. **A) **Phase contrast images of A2780s cells following 24 hrs of treatment with 5.0 μM M344 alone or in combination with 2 μg/ml cisplatin. **B) **A2780s cells subjected to combination platinum and M344 24 hr treatment demonstrates increased levels γH2A.X foci detected by immunoflourescence. Repeat of the experiment showed similar findings.

We further characterized the morphologic changes associated with combination treatment. Phase contrast images of A2780s cells are presented after 24 hrs of treatment in Figure 5A. Cells exposed to M344 and cisplatin showed characteristic features consistent with apoptosis, including cell rounding and detachment.

A hallmark of DNA double strand breaks, including those induced by cisplatin, is the formation of γH2A.X foci, resulting from the rapid phosphorylation of H2A.X at sites of DNA damage [28]. Following M344 +/- cisplatin treatment, A2780s cells were evaluated for γH2A.X foci formation using direct immunofluorescence (Figure 5B). Cells treated with DMSO control did not display γH2A.X foci and there was minimal γH2A.X foci formation with exposure of 5 μM M344 for 24 hrs. These findings suggest that treatment with single agent HDAC inhibitor was not sufficient to induce significant DNA damage. As expected, the majority of cells displayed many foci when treated with cisplatin alone. However, the addition of M344 to cisplatin resulted in a greater intensity of γH2A.X staining, which likely reflects an increase in DNA double strand breaks. Treated cells were also sorted via flow cytometry after being incubated with a fluorescent-labeled anti-γH2A.X antibody (Additional File 1). Treatment with the M344/cisplatin combination compared to cisplatin alone resulted in a greater percentage of cells with labeled γH2A.X.

### Decreased acetylated Histone 4 at the *BRCA1 *proximal promoter region following M344 treatment

**Figure 6:**
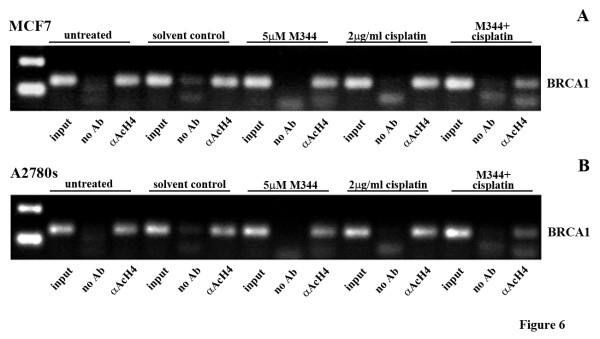
**ChIP analysis of the levels of acetylated histones at the BRCA1 promoter following M344 and cisplatin treatments**. **A) **MCF7 and **B) **A2780s cells treated with 5.0 μM M344 in combination with 2 μg/ml cisplatin for 24 hrs show reduced amounts of *BRCA1 *promoter DNA bound to acetylated histone 4 (AcH4). Real-time PCR products were run on a 1.6% agarose gel. "Input" controls: untreated; "No Ab" controls: incubation with agarose beads in the absence of αAcH4; and "αAcH4": incubation with agarose beads and αAcH4. The experiment was repeated twice with similar results.

A ChIP assay was performed in order to investigate whether M344 causes a direct change in *BRCA1 *gene expression by modulation of the chromatin structure of the *BRCA1 *promoter. MCF7 and A2780s cells were treated for 24 hrs with M344 (5 μM) and cisplatin (2 μg/ml), both individually, and in combination (Figure 6 and Additional File 2). With cisplatin treatment, there was an increase in *BRCA1 *DNA bound to acetylated histones. This supports previous reports that an increase in BRCA1 expression is reflective of the activation of the DNA damage response triggered by platinum agents [29]. The amount of *BRCA1 *DNA bound to acetylated histones decreased with the addition of this HDAC inhibitor to cisplatin, indicating that transcriptional repression may also be occurring in the combination treatment consistent with the RT-PCR and Western blot data in Figures 2 and 3.

## Discussion

BRCA1-deficient tumors have been shown to be more responsive to platinum-based chemotherapy, but as of yet, there is no molecular target of BRCA1 that can potentiate platinum sensitivity in OC patients. Prior work in our lab has demonstrated that co-treatment of OC cells, A2780s/cp, with the HDAC inhibitor M344 enhanced sensitivity to cisplatin [7]. In the present study, we further validate this finding in select breast and OC cell lines that differentially express BRCA1. The platinum-sensitive breast (MCF7) and OC (A2780s) cell lines, which displayed relatively high BRCA1 protein levels, displayed significant potentiation of cisplatin cytotoxicity in association with a reduction of BRCA1 protein with the addition of M344. Tumor cell lines with relatively low levels of BRCA1 protein (HCC1937, OVCAR-4) displayed inherent platinum sensitivity, and no significant enhancement of cisplatin was observed with the addition of the HDAC inhibitor. T-47D and A2780cp, cell lines known to be resistant to cisplatin, also elicited enhanced cytotoxicity of cisplatin with the addition of M344 in association with down regulation of BRCA1 protein, suggesting the potential of HDAC inhibition to enhance platinum sensitivity through a BRCA1-mediated mechanism.

The present study supports work by Burkitt and Ljungman [30], which showed that the HDAC inhibitor phenylbutyrate sensitized cisplatin-resistant head and neck cancer cell lines to cisplatin mediated by the abrogation of the Fanconi anemia/BRCA pathway. Phenylbutyrate was found to inhibit the formation of FANCD2 nuclear foci in conjunction with cisplatin and this correlated with down regulation of BRCA1. Furthermore, Zhang's group demonstrated that trichostatin A exposure delayed DNA damage repair in response to ionizing radiation by the suppression of key genes including *BRCA1 *[31]. A recent study by Kachhap *et al*. showed that valproic acid potentiated the sensitivity of prostate cancer cells to cisplatin through down regulation of HR repair and DNA damage response genes such as *BRCA1 *[32]. The decrease in *BRCA1 *gene transcription was due to a reduction in binding of the activating protein, E2F1, to the *BRCA1 *promoter. In the same prostate cancer cell line model, a new HDAC inhibitor, H6CAHA, suppressed the expression of BRCA1 mRNA, and when used in combination with γ-radiation, prevented the growth of tumor xenografts [33].

The sensitizing properties of HDAC inhibitors to DNA damaging agents has been linked to aberrant double strand break repair and cellular stress signaling [34].The present study confirms reports that HDAC inhibition, in combination with DNA damaging agents, increases the phosphorylation of H2A.X, a known marker of DNA double strand breaks [35-37]. A study conducted in a metastatic breast cancer cell line provides evidence of increased phosphorylation of H2A.X and enhanced sensitivity to vorinostat in combination with radiation [38]. In both human glioma and prostate cancer cells, vorinostat reduced DNA-dependent protein kinase (DNA-PK) and Rad 51, two critical components of DNA double strand break repair machinery [39]. In the human melanoma cell line, A375, vorinostat sensitized cells to radiation-induced apoptosis by inhibiting key DNA repair genes, Ku70, Ku80 and Rad 50 [37].Using cDNA expression arrays, phenylbutyrate attenuated the expression of DNA-PK and worked synergistically with ionizing radiation to induce apoptosis in prostate cancer cell lines [34].

BRCA1 has many diverse functions in the cell including transcriptional control through modulation of chromatin structure as BRCA1 is known to interact with the SWI/SNF chromatin remodeling complex [40]. The BRCA1-SWI/SNF complex is believed to be essential for the activation of genes involved in the DNA damage response and this complex has a direct role in HR by enabling access to sites of DNA damage. The BRCA1 C-terminal (BRCT) domain of the BRCA1 protein associates with both HDAC1 and HDAC2, and prior studies suggest that this association directly represses transcription [41]. In this study, the ChIP assay demonstrated that the amount of *BRCA1 *promoter DNA containing acetylated histones was decreased following M344 and cisplatin combination treatment relative to controls. This result suggests that *BRCA1 *is not a direct target of M344 activity, but that M344 may enhance the expression or activity of a transcriptional repressor of *BRCA1*. As an example, the Inhibitor of DNA binding (ID)-4 is a dominant negative transcriptional regulator [42], which has been shown to repress the *BRCA1 *promoter [43]. Studies have identified an inverse correlation between ID4 and BRCA1 mRNA and protein expression levels in breast and ovarian tumour tissue [44,45]. Further studies are needed to evaluate ID4's role in BRCA1 transcriptional activity and as a potential marker of BRCA1 expression.

Both *in vitro *and *in vivo *studies have demonstrated cytotoxic efficacy of single agent HDAC inhibitors in OC [14,20,46,47] and breast cancer [48,49] cell models. In our study, increasing doses of the HDAC inhibitor M344 down regulated BRCA1 protein expression in all cell lines examined except for the highest dose in MCF7 breast cancer cells. This could be due to a negative feedback loop involving the BRCA1 and HDAC1 proteins complexing with CtBP (C terminal-binding protein) on the BRCA1 promoter to inhibit its transcription [50]. A significant alteration in HDAC1 function and BRCA1 protein levels by the HDAC inhibitor M344 could alleviate the repression and cause an upregulation of BRCA1 transcription and subsequent protein expression. Since there is limited data in breast and ovarian cancer, studies conducted in other tumor cell models suggest the combination of HDAC inhibitors and DNA-targeted agents is a rational therapeutic approach in the treatment of OC. In the human oral squamous cell carcinoma cell line, HSC-3, SAHA enhanced cisplatin-induced apoptosis [51]. The study by Chen et al. [52] demonstrated a histone deacetylation-independent mechanism whereby HDAC inhibitors sensitized prostate cancer cell lines to DNA-damaging chemotherapeutic drugs, bleomycin, doxorubicin and etoposide. In their study, pretreatment of prostate cancer cells with HDAC inhibitors led to increased acetylation of Ku70 and impaired Ku70 function in repairing DNA double strand breaks resulting in enhance cell killing via a DNA repair-mediated mechanism. The HDAC inhibitor, PCI-24781, after treatment of Hodgkin and non-Hodgkin lymphoma cells with a PARP inhibitor, resulted in a synergistic increase in apoptosis and a decrease in RAD51 expression [53].

Recent clinical trials have evaluated HDAC inhibitors in solid tumors, both as a single agent and in combination with chemotherapy. A phase II study conducted by the Gynecologic Oncology Group, examined oral vorinostat in the treatment of persistent or recurrent epithelial ovarian or primary peritoneal carcinoma in patients who were platinum resistant/refractory (progression-free interval < 12 months) [16]. In the twenty-seven women enrolled, the incidence of significant toxicity was low, but only two had a progression-free interval over 6 months. A better response was seen in a phase II study combining valproic acid, the demethylating agent hydralazine, and chemotherapy in various resistant solid tumors including breast and ovarian cancer [54]. Twelve of fifteen patients (80%) overcame resistance to chemotherapy and showed either partial response or stable disease, although some hematologic toxicity was observed. A phase I study of vorinostat in combination with carboplatin and paclitaxel for advanced solid malignancies showed that the oral drug was well tolerated with eleven and seven of twenty-five patients analyzed demonstrating a partial response and stable disease, respectively, and encouraging anticancer activity in patients with previously untreated NSCLC [19]. A Phase I/II study of paclitaxel plus carboplatin in combination with vorinostat is currently underway in Denmark for patients with advanced, recurrent, platinum-sensitive epithelial OC (http://ClinicalTrials.gov, Identifier: NCT00772798). Further trials with correlative studies focusing on the BRCA1 pathway are needed to define a subset of the patient population which is most responsive to HDAC inhibitors.

There are several limitations to this study which merit consideration. Firstly, we recognize that studying the mechanism of BRCA1 down regulation by an HDAC inhibitor exclusively in cancer cell lines provides limited data that requires further exploration in an *in vivo *model. This will allow the involvement of extracellular components, such as the hormone estrogen, which has been shown to play a role in BRCA1 function [55]. Secondly, we and others have observed a lack of correlation between the BRCA1 mRNA and protein levels. This can be partly explained by the expression level of BRCA1 which oscillates with the cell-cycle and is regulated by both transcription and protein stability [56]. BRCA1 protein can be degraded by BARD1 in S-phase through the ubiquitin proteolysis pathway, thus unbalancing the mRNA to protein ratio [57]. Discrepancies between BRCA1 mRNA and protein can also be due to experimental limitations. Western blot analysis using the C-terminal BRCA1 antibody captures all splice variants of the gene [58] but is unable to detect truncated forms [59]. Furthermore, *BRCA1 *Δ11b, a splice variant abundantly expressed in many cells,[60] is not captured by the primers designed to cross the exon 11-12 boundary, which are used to measure mRNA levels by RT-PCR in our study. Thirdly, we propose that the enhanced sensitivity to cisplatin seen by HDAC inhibition is mediated though a BRCA1 mechanism although we are unable to provide direct evidence for this correlation. However, there is evidence in other reports that BRCA1 plays an essential role in inducing apoptosis in response to DNA-damaging agents in breast cancer cell line models. Inhibiting BRCA1 protein in MCF-7 cells increased cisplatin sensitivity [29] and depleted BRCA1 protein expression by siRNA-inhibited activation of the apoptotic pathway in response to DNA-damaging treatment [6]. Furthermore, *BRCA1 *transcription is known to be activated by the transcription factor E2F1 [61]. E2F1 protein levels were depleted with valproic acid exposure in prostate cancer cell lines and valproic acid reduced E2F1 binding to the BRCA1 promoter, thus providing insight into a mechanism for the down regulation of the *BRCA1 *gene by HDAC inhibition.

This study suggests that treatment with an HDAC inhibitor enhances the cytotoxicity of cisplatin therapy in ovarian and breast cancer cells and that this increased sensitivity may be mediated by a BRCA1 mechanism. The potentiation of platinum with an HDAC inhibitor may be a novel therapeutic option for advanced or recurrent OC patients with tumors expressing significant levels of BRCA1.

## Abbreviations

OC: ovarian cancer; HDAC: histone deacetylase; BRCA1: Breast Cancer 1; HR: homologous recombination.

## Competing interests

The authors declare that they have no competing interests.

## Authors' contributions

AMO, KDG and KVC carried out the molecular and cell viability studies and participated in the design of the study. AMO and NN helped to draft the manuscript. JIW and JD conceived of the study, participated in its design and coordination and helped to draft the manuscript. All authors read and approved the final manuscript.

## Supplementary Material

Additional file 1 **Evaluating the effect of M344, cisplatin and their combination on the γH2A.X expression by FACS analysis**. A2780s cells were treated with 1 μM M344, 2 μg/ml of cisplatin alone, and in combination, for 24 hrs. Cells were labelled with an anti-γH2A.X antibody and evaluated for fluorescent levels by flow cytometry that showed an increase in the proportion of cells with increased γH2A.X staining in the combination. Click here for file

Additional file 2 **ChIP analysis of the levels of acetylated histones at the p21 promoter following M344 and cisplatin treatments**. MCF7 cells treated with 5.0 μM M344 and 2 μg/ml cisplatin alone, and in combination for 24 hrs were subjected to ChIP analysis. AcH4 antibody was used for the immunoprecipitation. p21 promoter primers were used to quantify the DNA by PCR, which was then normalized to Input controls. Values represent the mean +/- SEM of two separate experiments. Click here for file
