# Supplementary figures and images for: The effect of the histone deacetylase inhibitor M344 on BRCA1 expression in breast and ovarian cancer cells
Source: Cancer Cell Int. 2011 Aug 19;11:29. doi: 10.1186/1475-2867-11-29 (PMC3175148; doi:10.1186/1475-2867-11-29)

## Slide 1
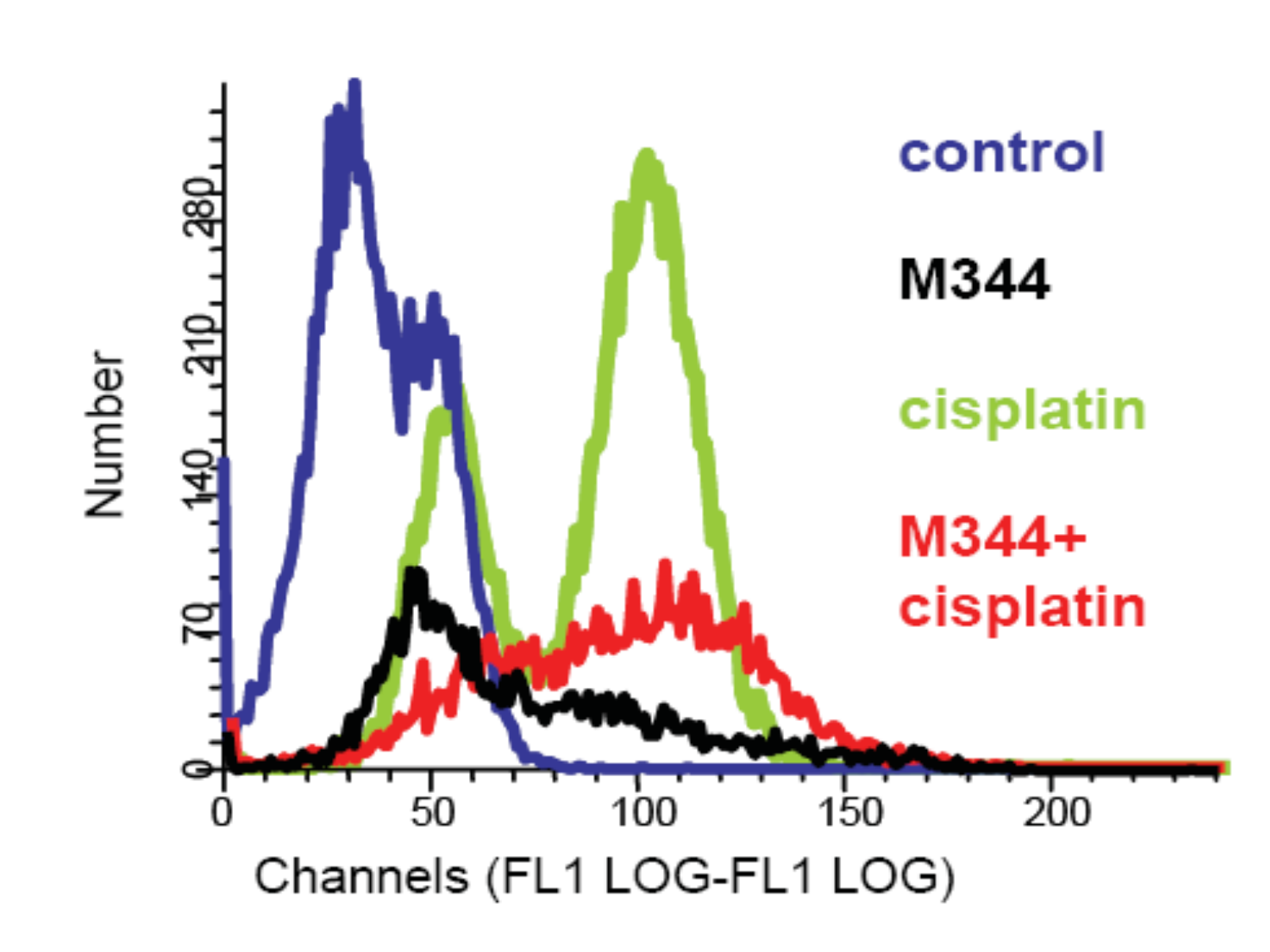

Supplement: Additional file 1 — Evaluating the effect of M344, cisplatin and their combination on the γH2A.X expression by FACS analysis. A2780s cells were treated with 1 μM M344, 2 μg/ml of cisplatin alone, and in combination, for 24 hrs. Cells were labelled with an anti-γH2A.X antibody and evaluated for fluorescent levels by flow cytometry that showed an increase in the proportion of cells with increased γH2A.X staining in the combination. [file 1475-2867-11-29-S1.PPT]

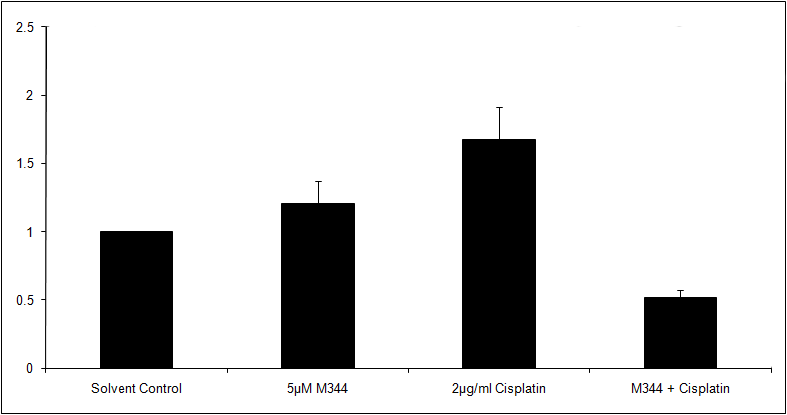

Supplement: Additional file 2 — ChIP analysis of the levels of acetylated histones at the p21 promoter following M344 and cisplatin treatments. MCF7 cells treated with 5.0 μM M344 and 2 μg/ml cisplatin alone, and in combination for 24 hrs were subjected to ChIP analysis. AcH4 antibody was used for the immunoprecipitation. p21 promoter primers were used to quantify the DNA by PCR, which was then normalized to Input controls. Values represent the mean +/- SEM of two separate experiments. [file 1475-2867-11-29-S2.TIFF]
